# Exploring the role of mindfulness on obligatory exercise among young athletes: mediating roles of obsessive passion and cognitive state anxiety

**DOI:** 10.3389/fpubh.2024.1381983

**Published:** 2024-05-01

**Authors:** Qianyuan Li, Li Li, Qianqian He, Huilin Wang

**Affiliations:** ^1^School of Physical Education, Hunan University of Science and Technology, Xiangtan, China; ^2^Faculty of Economics, Chulalongkorn University, Bangkok, Thailand; ^3^School of Business, Hunan University of Science and Technology, Xiangtan, China; ^4^Moray House School of Education and Sport, The University of Edinburgh, Edinburgh, United Kingdom

**Keywords:** young athletes, mindfulness, obsessive passion, cognitive state anxiety, obligatory exercise

## Abstract

**Introduction:**

In the current trend toward youthfulness and age reduction in competitive sports, the issue of obligatory exercise among young athletes is becoming more severe. This not only affects their physical and mental health but also hampers their future prospects in the sports world. While delving into the impact of mindfulness on the issue of obligatory exercise among young athletes, it reveals the mediating role of obsessive passion and cognitive state anxiety.

**Methods:**

This study is a cross-sectional research that employs convenience and snowball sampling methods. We selected 403 young athletes from several universities and high-level sports teams in the central-southern region of China as valid samples and used AMOS v.23 to construct a structural equation model to validate the hypotheses.

**Results:**

The research findings indicate a significant positive correlation between obsessive passion, cognitive state anxiety, and obligatory exercise. Furthermore, obsessive passion and cognitive state anxiety mediate the relationship between mindfulness and obligatory exercise. This implies that young athletes can better regulate their emotional state during training, manage training loads sensibly, and avoid issues with obligatory exercise through mindfulness training.

**Discussion:**

In conclusion, to enhance the cognitive levels of young athletes and reduce their obligatory exercise behaviors, national sports authorities and coaching teams should develop reasonable mindfulness training programs for athletes and encourage their participation in mindfulness training.

## 1 Introduction

Participating in high-intensity competitive sports can provide young athletes with positive physiological impacts and advanced athletic skills (1). However, the rigorous training intensity and the high-pressure competitive environment can also harm their behavioral health levels (2). In fact, away from the glamor of competition, one in every three to four athletes suffers from overtraining syndrome (3). Alarmingly, this issue is more severe among young athletes, as their eagerness to reach the top ranks of professional athletes leads them to adopt a more aggressive attitude toward training, pushing themselves beyond their psychological and physiological limits (4).

Recent trends indicate a rising number of student athletes and an increase in sports specialization and competitiveness, which has further exacerbated the issue of overtraining among young athletes (5, 6). Studies highlight that the training load for student athletes now surpasses that of past Olympic champions (7), with nearly half of young athletes’ sports injuries being attributable to obligatory exercise. Such practices not only risk their immediate health but also threaten to prematurely end their sports careers (8). Alarmingly, without early intervention, the rate of sports injuries related to obligatory exercise spikes threefold as athletes transition from high school to university (9), presenting a clear and urgent need for effective prevention and treatment strategies.

Mindfulness emerges as a vital tool in this context, offering a way to navigate the mental and physical challenges of competitive sports. Its importance lies in its ability to enhance athletes’ awareness of their bodies, thoughts, and emotions, fostering a more balanced approach to training and competition. Despite the significance of this practice, research on the role of mindfulness in combating the issues related to obligatory exercise, particularly among young athletes, remains sparse. Existing studies have primarily focused on identifying the precursors of obligatory exercise, such as socio-cultural influences (10), body dissatisfaction (11), emotional regulation styles (12), perfectionism (13), and sports ideals (14), as well as on measuring the overtraining problem among young athletes (15). Although the academic community has conducted extensive empirical analysis on obligatory training at both the cognitive (16) and behavioral levels (17), there is a lack of research discussing the connection between cognitive and behavioral aspects.

Mindfulness encourages athletes to calmly observe their current training experience without judgment (18), enhancing their focus and sense of control over the activity, and helping them concentrate during training to avoid additional anxiety (19). It also promotes a continuum from self-regulation to self-exploration and ultimately to self-liberation (20), allowing athletes to face previous unpleasant training experiences with equanimity, strengthening willpower, and avoiding negative expectations in cognitive anxiety (21), which has a positive effect on athletes’ sports-specific anxiety and competitive state anxiety (22–24). Furthermore, the potential mechanisms of self-regulation and behavioral flexibility in mindfulness training can shape athletes’ abilities in self-regulation and emotional regulation, improving adaptability to the environment and better managing emotions such as anger and restlessness (25–27). Additionally, since obsessive passion is an external manifestation of internalized stress, the open and accepting attitude fostered by mindfulness can help student athletes approach training mistakes with a tolerant mindset, thereby reducing stress at its root (28). Therefore, mindfulness is considered an effective method for managing the internal stress of athletes. Young athletes who undergo mindfulness training are more likely to achieve a relaxed state, avoiding the stress and burnout associated with competitive sports (29, 30).

Cognitive state anxiety is defined as the negative prediction and cognitive concern about one’s own sports abilities and status (31), reflecting to some extent the difficulty athletes face in coping with the immense pressure of high-level sports competition. Compared to low-level athletes, cognitive state anxiety has a greater impact on high-level athletes (32, 33). Affected by cognitive state anxiety, athletes experiencing negative self-doubt may become distracted, leading to incorrect judgments in subsequent training or competitions and, consequently, to the adoption of incorrect approaches to activities (34). Additionally, persistent criticism from coaches and teammates can also cause young athletes to remain in a state of anxiety about their body shape and sports level for a long time, leading to symptoms of obligatory training (35, 36). Obsessive passion, originating from the dualistic model of passion (37), is an emotion where an individual has a strong inclination to pursue and is willing to devote a significant amount of time and energy to it (38). Moreover, obsessive passion is more like an uncontrollable impulse, compelling athletes to sacrifice their personal health and stubbornly persist in training tasks that may improve their sports performance abilities, even in the face of a clear risk of injury (39–42). Furthermore, when individuals frequently fall into anxiety and remain immersed in this negative state for a long period, it can provoke anger and accompany reckless and impulsive behaviors (43), and individuals with strong emotional impulsiveness are more likely to have high levels of anxiety internalization symptoms (44, 45).

Unlike previous studies, this study focuses on the problem of obligatory exercise among young athletes, considering the potential impact of cognitive aspects on behavioral aspects, and proposes mindfulness as an intervention to improve the problem of obligatory training among young athletes. Although mindfulness has potential benefits for young athletes’ obsessive passion, cognitive state anxiety, and obligatory training, our understanding of how these factors are interconnected remains limited. The purpose of this study is to explore the potential mechanisms that form the relationships between these cognitive and behavioral factors and to determine strategies for improving the problem of obligatory exercise among athletes.

Based on the above, this study proposes the following hypotheses:

*H1*: Mindfulness has a negative and significant impact on cognitive state anxiety.

*H2*: Mindfulness has a negative and significant impact on obsessive passion.

*H3*: Cognitive state anxiety has a positive and significant impact on obsessive passion.

*H4*: Cognitive state anxiety has a positive and significant impact on obligatory exercise.

*H5*: Obsessive passion has a positive and significant impact on obligatory exercise.

*H6*: Cognitive state anxiety and obsessive passion mediate the relationship between mindfulness and obligatory exercise.

**Figure 1:**
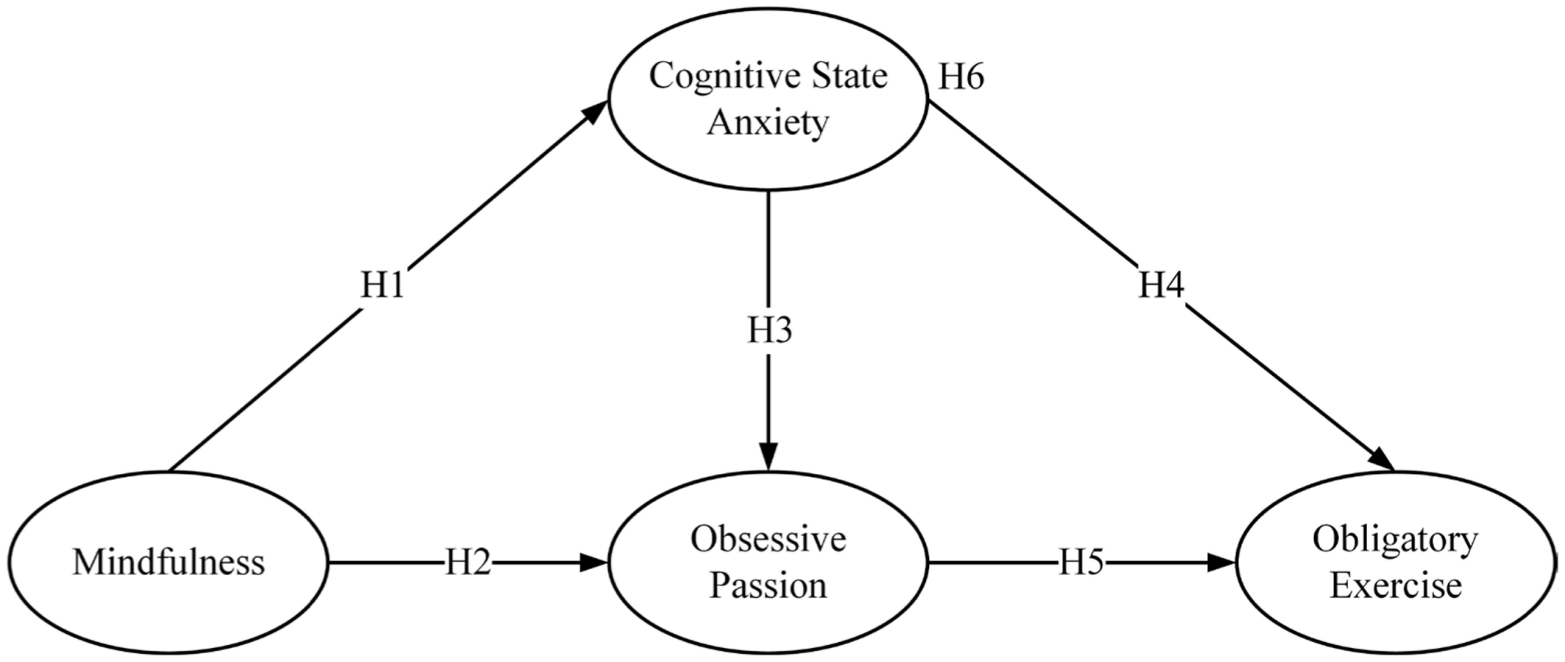
Hypothesis model.

All hypotheses are summarized in Figure 1.

Therefore, this study aims to: (1) understand the obligatory exercise issues among young Chinese athletes; (2) discuss the relationships between mindfulness, cognitive state anxiety, obsessive passion, and obligatory exercise in young athletes; and (3) provide recommendations for preventing and intervening in obsessive training behaviors for sports education institutions, including the National Sports Administration, universities, professional sports schools, and sports clubs.

## 2 Methodology

### 2.1 Participants and procedures

This study conducted a web-based questionnaire survey among high-level young athletes. To collect research data, the researchers employed convenience sampling and snowball sampling methods. After obtaining approval from local authorities, the researchers reached out to athletes from four universities, youth training centers, and high-level clubs in different provinces of central and southern China. Using social media platforms such as QQ and WeChat, the researchers inquired about their interest in participating in the study and sent the questionnaire tool link, inviting them to participate sincerely. All participants were informed of the survey’s purpose and volunteered before completing the questionnaire. Additionally, based on the snowball method, athletes who completed the questionnaire were encouraged to invite their friends and teammates to participate in the survey. Participants who completed the survey were then given training equipment as a token of appreciation. By the end of July, we had distributed 600 questionnaires and successfully collected responses from 482 athletes. After eliminating invalid questionnaires (due to data anomalies, missing information, blank responses, etc.), a total of 403 valid questionnaires were obtained, resulting in a valid response rate of 62.7%.

Table 1 presents the demographic characteristics of the 403 student athletes participating in this survey. Among the respondents, (1) the male-to-female ratio was approximately two to one; (2) 69% of the participants were under the age of 23; (3) athletes participating in badminton, soccer, basketball, and track and field accounted for half of the total; (4) in the past year, about 70% of athletes participated in sports competitions only 1–3 times. The reason for this relatively high number is attributed to the official lifting of COVID-19 prevention and control measures in China on January 8, 2023, allowing sports competitions to resume. Prior to that date, sports competitions could not be held normally, and athletes were unable to participate in them.

**Table 1:** Demographic characteristics of participants.

Profiles	Survey (%)
**Gender**	
Male	260 (64.5%)
Female	143 (35.5%)
**Age**	
14–17	35 (8.7%)
18–20	97 (24.1%)
21–23	146 (36.2%)
>23	125 (31.0%)
**Sports items**	
Ball sports	195 (48.4%)
Track and field sports	70 (17.4%)
Other sports	138 (34.2%)
**The number of sports competitions participated in the past year**	
1–3	275 (68.2%)
4–6	59 (14.7%)
7–9	23 (5.7%)
≥10	46 (11.4%)

### 2.2 Instruments

The questionnaire comprised five sections. The first section requested respondents to provide their demographic information, including age, gender, the sports they were engaged in, the number of competitions participated in the past year, and the duration of training cessation due to sports injuries or illnesses in the past year.

The second section utilized five items from the scale developed by Feldman et al. (46) to collect relevant data on respondents’ mindfulness levels. Sample items included “I try to pay attention to my thoughts without judging them.” The third section employed four items from the scale developed by Lundqvist and Hassmén (47) to gather data on respondents’ cognitive state anxiety. Sample items included “I worry about my performance when I am engaged in sports.” The fourth section used four items from the scale developed by Vallerand et al. (37) to collect data on respondents’ obsessive passion. Sample items included “There is a strong impulse to engage in this sport that I cannot resist.” The fifth section utilized five items from the scale developed by Steffen and Brehm (48) to compile data on respondents’ obligatory exercise. Sample items included “I feel guilty when I do not exercise.” All four scales were measured using a Likert five-point scale, with response options ranging from 1 (Strongly Disagree) to 5 (Strongly Agree).

The researchers adapted some items in the scales to better suit the habits and context of Chinese individuals, as well as to ensure the scale items were appropriate for our research context. Please refer to Table 2 for the content of the scales. A pilot test was conducted in advance to ensure its reliability (49). A total of 96 valid questionnaires were anticipated, and the results indicated that Cronbach’s alpha coefficients were all above 0.8, confirming the researchers’ modifications to the scales based on the context were justified.

**Table 2:** Reliability and validity.

Items	Loadings	Cα	CR	AVE
**Mindfulness (MI)**		0.943	0.944	0.770
MI1: It is easy for me to concentrate on what I am doing	0.882			
MI2: I can accept things I cannot change	0.841			
MI3: I can usually describe how I feel at the moment in considerable detail	0.899			
MI4: I try to notice my thoughts without judging them	0.879			
MI5: I am able to pay close attention to one thing for a long period of time	0.886			
**Cognitive State Anxiety (CS)**		0.885	0.883	0.654
CS1: I am concerned about choking under pressure	0.751			
CS2: I am concerned about I am concerned that others	0.757			
CS3: I am concerned about will be disappointed with	0.878			
CS4: I am concerned about my performance	0.842			
**Obsessive Passion (OP)**		0.938	0.939	0.793
OP1: I cannot live without sports	0.897			
OP2: The urge to engage in sports is so strong. I cannot help myself from participating in this activity	0.892			
OP3: I have difficulty imagining my life without sports	0.908			
OP4: I am emotionally dependent on sports	0.864			
**Obligatory Exercise (OE)**		0.892	0.892	0.624
OE1: When I miss an exercise session, I feel concerned about my body possibly getting out of shape	0.767			
OE2: When I do not exercise, I feel guilty	0.813			
OE3: I frequently “push myself to the limits”	0.758			
OE4: I have had daydreams about exercising	0.818			
OE5: Sometimes I find that my mind wanders to thoughts about exercising	0.791			

### 2.3 Data analysis

In this study, AMOS v.23 was employed to construct a structural equation model (SEM) to examine how athletes, through mindfulness meditation, reduce cognitive state anxiety and obsessive passion, thereby improving their tendency toward obligatory exercise. The maximum likelihood (ML) estimation method was utilized to estimate the model parameters. A two-step modeling approach was employed to assess both the measurement and structural models. First, a comprehensive evaluation of the model’s reliability and validity was conducted. Subsequently, fit indices and path coefficients of the hypothesis model were measured, and the presence of mediating effects was examined.

To mitigate potential common method variance (CMV) arising from self-reported behaviors, researchers followed the recommended approach outlined by Mossholder et al. (50). As part of this approach, a comparative analysis was conducted between model one and model two, focusing on variations in degrees of freedom and chi-square values. The results indicated that the chi-square value for model one was 3078.434, encompassing 135 degrees of freedom and yielding a *p*-value below 0.001. Similarly, for model two, the chi-square value was 252.106, accompanied by 129 degrees of freedom and a *p*-value below 0.001. These findings affirm the congruence of fit between model one and model two, suggesting that CMV is not a concern within the context of this study.

## 3 Results

### 3.1 Measurement model

The reliability and validity assessment of latent variables incorporated confirmatory factor analysis (CFA) using AMOS v.23. Table 2 displays the factor loadings, Cronbach’s α coefficients, composite reliability (CR), and average variance extracted (AVE) for each item on the scale. As shown in Table 2, all variables demonstrated Cronbach’s α values exceeding 0.8, affirming robust internal consistency within the model structure as guided by Fornell and Larcker (51). Additionally, the AVE for each variable exceeded 0.6 (as noted in Table 2), surpassing the minimal acceptable threshold of 0.5. Furthermore, the CR of each latent variable surpassed 0.8, underscoring the model’s robust convergent validity. The resilience of convergent validity across proposed models was well-established. Factor loadings from principal component factor analysis ranged from 0.751 to 0.908, reinforcing the measurement model’s robust construct validity. Table 3 shows that the square root of the AVE is on the diagonals, while the off diagonals are Pearson’s correlations of constructs. The square root of the AVE on the diagonals exceeds the inter-construct correlations, confirming discriminant validity.

**Table 3:** Pearson correlation.

**Construct**	**MI**	**CS**	**OP**	**OE**
MI	**(0.877)**			
CS	−0.354**	**(0.809)**		
OP	−0.349**	0.558**	**(0.891)**	
OE	−0.208**	0.616**	0.563**	**(0.789)**

The square root of the AVE is in diagonals; off diagonals are a Person’s corrections of contracts. **p < 0.01.

### 3.2 Structural model

**Figure 2:**
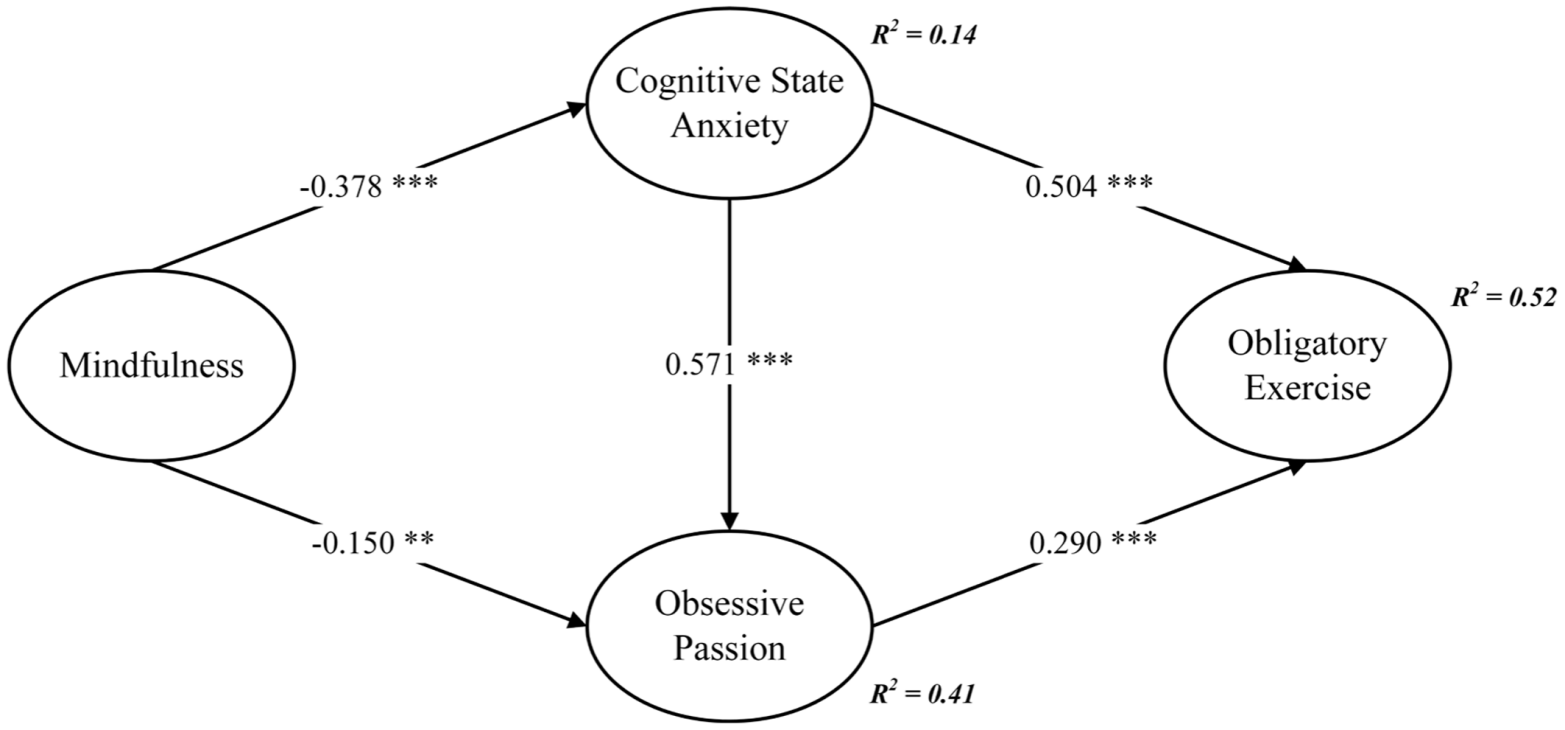
Structural path model. **p < 0.01, ***p < 0.001.

Following a comprehensive evaluation of the measurement model’s reliability and validity, this study advanced to scrutinize the structural model using AMOS v.23, aimed at substantiating the formulated hypotheses. Using 5,000 bootstrap samples, the outcomes of CFA consistently aligned with established criteria (χ^2^/df = 2.755, GFI = 0.907, NFI = 0.940, TLI = 0.954, CFI = 0.961, RMSEA = 0.066), unequivocally indicating an optimal fit between the model and empirical data. Moreover, the interconnectedness of variables was corroborated by the Pearson correlation results outlined in Table 3. The standardized coefficients for variables within the structural equation model are visually depicted in Figure 2.

As illustrated in Figure 2, mindfulness exhibited a direct and negative correlation with cognitive state anxiety (*β* = −0.378, *p* < 0.001) and a negative correlation with obsessive passion (*β* = −0.150, *p* < 0.01), effectively substantiating H1 and H2, respectively. Additionally, cognitive state anxiety showed a direct and positive correlation with obsessive passion (*β* = 0.571, *p* < 0.001), robustly supporting H3. Similarly, cognitive state anxiety had a direct and positive association with obligatory exercise (*β* = 0.504, *p* < 0.001), affirming H4. Notably, obsessive passion was identified as having a direct and positive correlation with obligatory exercise (*β* = 0.290, *p* < 0.001), substantiating H5.

The evaluation of mediating effects, as depicted in Table 4, was conducted using bootstrap estimation with 5,000 resamples and 95% bias-corrected confidence intervals. The findings distinctively highlighted the indirect effect of mindfulness on obligatory exercise, mediated through cognitive state anxiety and obsessive passion. This mediation yielded a robust estimate of −0.297 (SE = 0.041, CI = [−0.377, −0.213], *p* < 0.001), thus providing comprehensive support for H6.

**Table 4:** Standardized indirect effect.

	**Point estimate**	**Product of coefficients**		**Bootstrapping**		
				**Bias-Corrected 95% CI**		**Two-Tailed Significance**
		**SE**	***Z***	**Lower**	**Upper**	
MI → OE	−0.297	0.041	−7.244	−0.377	−0.213	0.000 (***)

***p < 0.001.

## 4 Discussion

### 4.1 Theoretical contributions

This study makes several theoretical contributions to the analysis of athletes’ obligatory exercise. Firstly, unlike existing research on athletes’ obligatory exercise that focuses on athletes’ body dissatisfaction (11) and socio-cultural factors (10), this study is pioneering in combining cognitive and behavioral aspects to explore the relationship between mindfulness and obligatory exercise among young athletes. The results reveal a significant negative correlation between mindfulness and cognitive state anxiety (see Figure 2), supporting the findings of Bühlmayer et al. (19). In our model, the impact of mindfulness on obligatory passion was the most pronounced, followed by cognitive state anxiety. These factors, cognitive state anxiety and obligatory passion, mediate the relationship between mindfulness and obligatory exercise, explaining 52% of the variance in obligatory exercise as depicted in Figure 2. This indicates that the prevalence of obligatory exercise issues among young athletes is intimately linked to how they manage anxiety and passion. Mindfulness training can potentially diminish the impacts of obligatory passion and cognitive state anxiety on their cognitive and judgment skills, thereby effectively reducing the occurrence of obligatory exercise issues. This insight provides a solid pathway and theoretical foundation for further research into the relationship between mindfulness training and obligatory exercise issues among young athletes.

### 4.2 Practical implications

Considering the positive impact of mindfulness on reducing athletes’ obsessive passion and cognitive state anxiety, as well as its indirect effect on alleviating obligatory exercise problems, the following recommendations are proposed: Firstly, at the national level, the National Sports Administration and its subordinate government departments should prioritize mental health in young athletes. Given their less mature mindset, athletes are more susceptible to cognitive distortions (4), making the incorporation of mindfulness training into daily plans crucial. Establishing a reasonable evaluation system and regularly inspecting the psychological conditions of athletes nationwide will provide a strong support system for their mental health. It is essential to collect and analyze data on the cognitive impact of mindfulness training methods on athletes, allowing for timely adjustments to ensure their effectiveness and applicability.

Secondly, at the level of athletes’ surroundings, including their team psychologists, coaches and training partners, team sports psychologists play a crucial role. They must promptly understand the latest research on the impact of mindfulness on athletes, encourage active participation in mindfulness training, and meticulously observe and record the athletes’ psychological states to maintain mental health. Coaches should identify athletes suffering from obligatory exercise issues and adjust training plans accordingly, in consultation with sports psychologists, while eliminating the unhealthy training atmosphere of “the more, the better.”

Finally, at the individual level, athletes should actively participate in mindfulness training and maintain good communication with coaches and teammates. When early symptoms such as anxiety and stress arise, athletes should provide honest feedback on their psychological issues (2) to prevent obligatory exercise problems. Early detection and treatment are crucial for realizing athletic potential.

For a long time, the focus has been on athletes’ performances, overlooking the negative impacts of long-term high-intensity training. Despite studies confirming that mindfulness training alleviates negative emotions like anxiety, tension, and fatigue (29) and positively impacts performance (52), its importance is often unrecognized by management organizations, coaches, and athletes themselves. Furthermore, influenced by personal self-esteem, many young athletes are reluctant to seek help for psychological issues such as training pressure and panic (53), leading to the loss of control over their training behavior. This study highlights the positive influence of cognitive improvements and encourages greater participation in mindfulness training. We recommend that young athletes in China try the Mindfulness-Acceptance-Insight-Commitment (MAIC) program, suited to the Chinese social context (54), to gain a comprehensive understanding of their training activities and reduce the occurrence of negative situations such as obligatory exercise.

### 4.3 Limitations

Firstly, the participants of our study were mainly drawn from universities and sports teams within the central and southern regions of China. Recognizing the need for a more diverse and representative sample, future studies should aim to extend the geographical scope and include a wider range of sports disciplines. This expansion would not only enhance the representativeness of the sample but also improve the generalizability of the research findings across different contexts. Secondly, given the cross-sectional nature of our study, which lacks a temporal dimension, we acknowledge the limitation this presents in understanding the dynamic nature of mindfulness training effects. Future research could benefit from adopting a longitudinal approach, allowing for a detailed examination of how the relationships between variables evolve over time and the sustained impacts of mindfulness training on athletes’ behavior and psychological well-being. Thirdly, our research concentrated on young competitive athletes, leaving a gap in our understanding of mindfulness training’s relevance for non-competitive or recreational athletes. Future inquiries should explore the applicability of our findings to these groups, taking care to address potential biases in self-reporting by participants. Fourthly, our study was limited by its exclusive reliance on quantitative research methods. Incorporating qualitative insights on top of quantitative data could significantly enrich our understanding of how athletes personally perceive the impact of mindfulness on their training and psychological states. Therefore, future research should aim to integrate qualitative methodologies, such as interviews or focus groups, to capture the nuanced perspectives of athletes regarding mindfulness practices. This mixed-methods approach would offer a more holistic view of the mindfulness training effects, providing deeper insights into the athletes’ subjective experiences and the complex interplay between mindfulness, training habits, and mental well-being.

## 5 Conclusion

In accordance with the research objectives, this study identifies mindfulness, obsessive passion, and cognitive state anxiety as crucial factors influencing obligatory exercise behavior in young athletes. Specifically, mindfulness can indirectly improve the obligatory exercise issues of young athletes by reducing obsessive passion and cognitive state anxiety, acting as two mediating variables. Therefore, this study recommends that athlete management organizations and coaches prioritize monitoring the mental health levels of athletes and integrate mindfulness training into their daily routines. Simultaneously, young athletes should proactively respond to external assistance regarding their psychological well-being, attempting to utilize mindfulness to control negative training emotions, ultimately reducing the occurrence of obligatory exercise problems.

## Data availability statement

The raw data supporting the conclusions of this article will be made available by the authors, without undue reservation.

## Ethics statement

The studies involving humans were approved by Ethics Committee of the School of Physical Education of Hunan University of Science and Technology (No. ECBPEHNUST 2022/0012). The studies were conducted in accordance with the local legislation and institutional requirements. The participants provided their written informed consent to participate in this study.

## Author contributions

QL: Conceptualization, Formal analysis, Investigation, Methodology, Writing – original draft, Writing – review & editing. LL: Funding acquisition, Investigation, Resources, Writing – original draft, Writing – review & editing. QH: Methodology, Validation, Writing – original draft, Writing – review & editing. HW: Conceptualization, Project administration, Supervision, Writing – original draft, Writing – review & editing.
